# Inside out: efflux of carbon dioxide from leaves represents more than leaf metabolism

**DOI:** 10.1093/jxb/erx155

**Published:** 2017-05-30

**Authors:** Samantha S Stutz, Jeremiah Anderson, Rachael Zulick, David T Hanson

**Affiliations:** Department of Biology, University of New Mexico, MSC03-2020, 1 University of New Mexico, Albuquerque, NM, USA

**Keywords:** *Brassica napus*, carbon cycle, CO_2_ efflux, internally transported CO_2_, leaf respiration, *Populus deltoides*, stem [CO_2_*], terrestrial carbon sink, tunable diode laser absorbance spectroscopy, xylem-transported CO_2_

## Abstract

High concentrations of inorganic carbon in the xylem, produced from root, stem, and branch respiration, travel via the transpiration stream and eventually exit the plant through distant tissues as CO_2_. Unlike previous studies that focused on the efflux of CO_2_ from roots and woody tissues, we focus on efflux from leaves and the potential effect on leaf respiration measurements. We labeled transported inorganic carbon, spanning reported xylem concentrations, with ^13^C and then manipulated transpiration rates in the dark in order to vary the rates of inorganic carbon supply to cut leaves from *Brassica napus* and *Populus deltoides*. We used tunable diode laser absorbance spectroscopy to directly measure the rate of gross ^13^CO_2_ efflux, derived from inorganic carbon supplied from outside of the leaf, relative to gross ^12^CO_2_ efflux generated from leaf cells. These experiemnts showed that ^13^CO_2_ efflux was dependent upon the rate of inorganic carbon supply to the leaf and the rate of transpiration. Our data show that the gross leaf efflux of xylem-transported CO_2_ is likely small in the dark when rates of transpiration are low. However, gross leaf efflux of xylem-transported CO_2_ could approach half the rate of leaf respiration in the light when transpiration rates and branch inorganic carbon concentrations are high, irrespective of the grossly different petiole morphologies in our experiment.

## Teaser

Through supplying ^13^C to leaves, the effect of the transportation of respired CO_2_ on measured leaf respiration is shown.

## Introduction

Environmental control of leaf-level respiration is poorly understood and the global flux of respired CO_2_ from leaves is massive. Current estimates indicate that global leaf respiration is 21–28 Pg C yr^−1^, which is approximately 3–4 times larger than all emissions from fossil fuel burning globally (Atkin *et al.*, 2007; Atkin *et al.*, 2014). The combination of uncertainty and small errors in the measurement of respiration can have large consequences (Hanson *et al.*, 2016). For example, a 1% error in predictions of leaf respiration, i.e. 0.21–0.28 Pg C yr^−1^, would account for about a third of the 0.7 Pg C yr^−1^ uncertainty in global carbon models (Canadell *et al.*, 2007). Such a large role of leaf respiration is consistent with analyses showing that behavior of the carbon cycle is the second greatest source of uncertainty in climate model predictions of global temperature. Over 15% of this uncertainty can be attributed to temperature feedbacks on respiration and net primary productivity (Bodman *et al.*, 2013). Coupled climate Earth System Models and Terrestrial Biosphere Models have made some advances that improve the modeling of respiration (King *et al.*, 2006; Atkin *et al.*, 2008; Wythers *et al.*, 2013; Atkin *et al.*, 2015; Wullschleger *et al.*, 2015), while also highlighting the need for more data (King *et al.*, 2006; Atkin *et al.*, 2008; Reich, 2010; Atkin *et al.*, 2015; Wullschleger *et al.*, 2015).

**Fig. 1.**
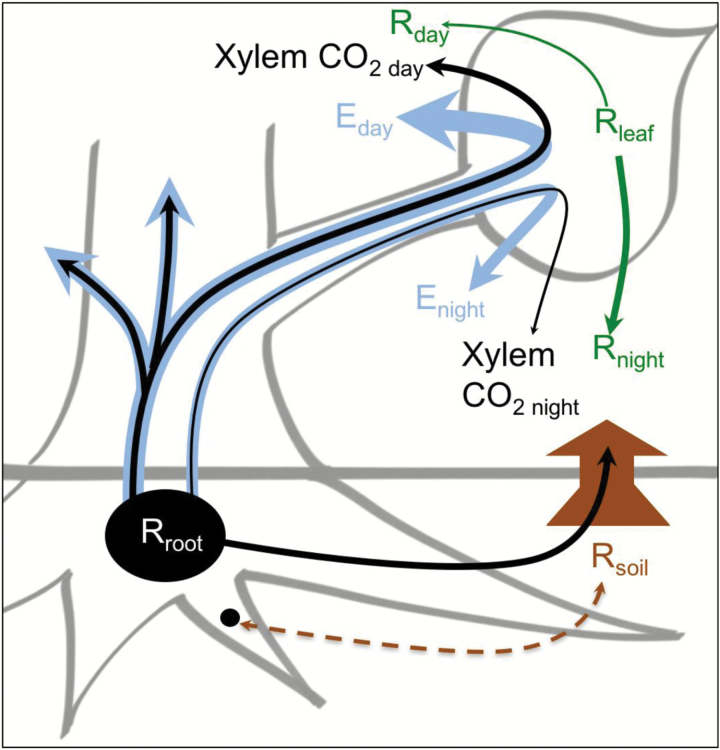
Conceptual illustration of relative fluxes for respiration, transpiration, and xylem-transported CO_2_ between day and night. Respiration in the root (R_root_) causes high concentrations of CO_2_ to accumulate in the roots and stem (large black oval) relative to the surroundings, while the accumulation should be much lower in smaller roots (small black circle) due to lower resistance to efflux. Some CO_2_ from R_root_ effluxes into the surrounding soil, joining the pool of soil respired CO_2_ (R_soil_). In small roots, some CO_2_ from R_soil_ could enter the root if the root CO_2_ concentration is lower than the soil concentration. A large fraction of the CO_2_ from R_root_ is transported up the stem in the transpiration stream (blue lines). During the day, transpiration is large (E_day_) and carries a large amount of xylem-dissolved CO_2_ to the leaf that can efflux into the atmosphere (xylem CO_2_ day) if not captured by photosynthesis. Respiration of leaf cells (green lines) in the day (R_day_) is thought to be much smaller than in the night (R_night_). However, in the night transpiration (E_night_) is much smaller than in the day (E_day_), so the efflux of xylem-dissolved CO_2_ in the night (xylem CO_2_ night) should be proportionately smaller than in the day (xylem CO_2_ day).

All methods for measuring or modeling the rate of gross non-photorespiratory CO_2_ efflux, i.e. respiration, from leaves implicitly assume that all CO_2_ efflux is derived from metabolism occurring in leaf cells (e.g. Farquhar *et al.*, 1980). Similarly, it has often been assumed that in roots, internally generated CO_2_ diffuses into the soil before diffusing into the atmosphere (Kuzyakov, 2006). However, studies with woody plants show that a large fraction of CO_2_ generated by root and stem respiration can be transported through the xylem, with efflux from the plant to the atmosphere through the stem and branches at a point remote from its production (Levy *et al.*, 1999; McGuire and Teskey, 2002; McGuire *et al.*, 2009; Aubrey and Teskey, 2009; Bloemen *et al.*, 2013*a*,b; Bloemen *et al.*, 2015*a*; Steppe *et al.*, 2015) (Fig. 1). The efflux of xylem-transported CO_2_ can be large because the total inorganic carbon concentration ([CO_2_*], the sum of [CO_2_]_aq_, [H_2_CO_3_], [HCO_3_^−^] and [CO_3_^2−^]) in the xylem ranges from ~0.05 mmol l^−1^ to ~13 mmol l^−1^ - levels that are ~30–750 times higher than if equilibrated with current atmospheric [CO_2_] (Teskey *et al.*, 2008). Previous studies have found xylem-transported CO_2_* is recycled by photosynthesis in corticular and woody stem tissue (Teskey *et al.*, 2008), branch tissue (McGuire and Teskey, 2002; McGuire *et al.*, 2009; Bloemen *et al.*, 2013*a*,b) and even leaves (Stringer and Kimmer, 1993; McGuire *et al.*, 2009; Bloemen *et al.*, 2013*a*,b; Bloemen *et al.*, 2015*a*). The relative magnitude of this recycling in each part of the tree was demonstrated by [Bibr CIT0013][Bibr CIT0013]). They added ^13^CO_2_* to the stems of *Populus deltoides* in the field under low (1.4 mmol l^−1^ [^13^CO_2_*]) and high (12 mmol l^−1^ [^13^CO_2_*]) treatments and used destructive sampling for mass balance calculations to track the fate of the labeled carbon. They concluded that 82.6% and 94.4% of ^13^CO_2_* added to the stems of under low and high treatments, respectively, was transported through the transpiration stream and diffused into the atmosphere through some combination of the stem and branches over a period of two days. Most of the ^13^CO_2_* retained by the plant was recycled by photosynthesis in the stem and branches, with a small fraction recycled by leaves; 2.7% and 0.5% under the low- and high-labels, respectively (Bloemen *et al.*, 2013b). Very little data exist to assess if the low rates of recapture of xylem-transported CO_2_ by leaves is the result of little xylem-transported CO_2_* reaching the leaves or because only a small amount of what reaches the leaves is captured before diffusing away. When supplying a solution of H^14^CO_3_^−^ in water to excised leaves, Stringer and Kimmerer (1993) estimated that 0.14% and 0.38% diffused out of the leaf as ^14^CO_2_ under light intensities of 660 μmol quanta m^−2^ s^−1^ and 70 μmol quanta m^−2^ s^−1^, respectively, and about 83% diffused out in the dark. The large fraction exiting leaves in the dark would appear as leaf respiration but would be under the control of transpiration and rates of respiration in the stem and roots. In the day, the gross flux would be much higher, so a substantial amount of CO_2_ derived from root and stem respiration could be used by leaves for photosynthesis or exit leaves and confound the measurement of leaf respiration in the light.

Unlike stem and root respiration, leaf respiration is thought to differ between the day and night because of a reduction in the cyclic nature of the Krebs Cycle when leaves are illuminated (Tcherkez *et al.*, 2009). However, this has only been demonstrated in *Xanthium strumarium*, so its application across species is uncertain. Measurements of the inhibition of leaf respiration in the light range widely from 16–77% (Atkin *et al.*, 2000; Hurry *et al.*, 2005; Ayub *et al.*, 2011).

Additionally, rates of leaf day and night respiration can respond differently to sustained drought, with day respiration inhibited by drought more than night respiration (Ayub *et al.*, 2011). In some cases the ratio of light to dark respiration increases with rising leaf temperature, through a reduction in the light suppression of respiration at high temperatures (Way *et al.*, 2015).

The objective of this study is to test the dynamic relationship among the [CO_2_*] in the xylem, the rate of transpiration and the rate of gross CO_2_ efflux in the woody plant *P. deltoides* and the herbaceous plant *Brassica napus*. We hypothesize that the efflux of xylem-transported CO_2_ from leaves could be large relative to respiration and will be controlled by [CO_2_*] and transpiration rate, with some effect of leaf and petiole morphology. Additionally, data on these fluxes should facilitate future efforts to create mechanistic models of the gross CO_2_ efflux from leaves in response to prevailing and predicted environmental conditions, filling in the gaps left by previous studies that have not looked at the efflux of xylem-transported CO_2_ from the leaves.

## Materials and Methods

### Growth conditions and plant propagation

*Brassica napus* (L. stellar DH GT060615) and *Populus deltoides* (W. Bartram ex Marshall) were grown under natural light in an unshaded greenhouse in February and March 2013 and 2014, with mid-day photosynthetically active radiation (PAR) at pot level of 1200 μmol m^−2^ s^−1^ at the University of New Mexico in Albuquerque, NM, under ambient CO_2_, 21°C/18°C and 24°C/21 °C day/night for *B. napus* and *P. deltoides*, respectively.

*B. napus* was started from seed and *P. deltoides* was clonally propagated by cuttings. *B. napus* was sown in 500 mL pots with Metro-Mix 300 potting soil (Sun Gro Horticulture, Seba Beach, AB, Canada). *P. deltoides* cuttings were initially placed in containers filled with Metro-Mix 300 potting soil and allowed to grow for approximately two months before the soil was washed off the roots and cuttings were transplanted into 3.8 L pots with a mixture of approximately two parts vermiculite (Therm-O-Rock West Inc., Chandler, AZ, USA) to one part perlite (Therm-O-Rock West Inc., Chandler, AZ, USA) and less than 5% Agrosoke crystals (Agrosoke International Arlington, TX, USA). *B. napus* and *P. deltoides* were fertilized twice weekly with Peters 20-20-20 fertilizer (Scotts Miracle-Gro, Marysville, OH, USA) and once weekly with chelated liquid iron (ferti-lome, Bonham, TX, USA). *B. napus* plants were measured between 14 and 25 days after geminating.

### Tunable diode laser absorbance spectroscopy and leaf gas exchange measurements

A LI-6400 (LI-COR Biosciences, Lincoln, NE, USA) was coupled to a tunable diode laser (TDL) absorbance spectrometer (model TGA 100; Campbell Scientific, Inc., Logan, UT, USA) to measure online ^12^CO_2_ and ^13^CO_2_ exchange. Isotope calibration consisted of a high and a low CO_2_ tank that spanned the expected range of [CO_2_] of each isotopologue for the LI-COR reference and sample (Barbour *et al.*, 2007). The TDL cycled between calibrations of the high and low CO_2_ tank along with the LI-COR reference, with sample line measuring for one minute at each site. However, only the last 10 seconds of data from each site were used for calculations via the TDL LI-COR processing package (Erhardt and Hanson, 2013) in R (R Core Development Team, 2011). The TDL measures [^12^CO_2_] and [^13^CO_2_], so the net fluxes of each were calculated as in normal gas exchange, where total [CO_2_] is measured in the air supplied to the leaf chamber and within the well-mixed leaf chamber. All data presented reflects the ^12^CO_2_ and ^13^CO_2_ effluxes measured with the TDL.

The entire, highest, fully expanded *B. napus* leaf, with a petiole length between 6.5 cm and 8.5 cm, or a fully expanded *P. deltoides* leaf, with a petiole length between 4 cm and 7 cm, was placed in a large 80 cm^2^ custom clear topped chamber attached to a LI-6400 (LI-COR Biosciences, Lincoln, NE, USA). An RGB LED light source (LI-COR Biosciences) set at 1200 μmol quanta m^−2^ s^−1^ or 1500 μmol quanta m^−2^ s^−1^ for *B. napus* and *P. deltoides*, respectively, was placed over the custom LI-6400 leaf chamber with a leaf temperature of 25°C and 3.8 Pa (380 μmol mol^−1^) CO_2_ reference. The leaves were photographed and the projected leaf area was estimated using ImageJ (US National Institutes of Health, Bethesda, MD, USA).

Once photosynthesis reached a steady state, the leaf was detached from the plant and the petiole was placed in a 40 mmol l^−1^ KCl solution. The detached leaf remained in the KCl solution for ~10 minutes while rates of transpiration and photosynthesis were monitored. Photosynthesis did not change when the leaf was cut, while transpiration increased by ~0.2 mmol H_2_O m^−2^ s^−1^. The KCl solution was swapped for a solution containing 99 atom % ^13^C sodium bicarbonate dissolved in 40 mmol l^−1^ KCl at one of three [^13^CO_2_*]: 1.19, 5.95, or 11.9 mmol l^−1^. Individual leaves were only provided a single [^13^CO_2_*]. These concentrations span the range of observed values from previous field studies for tree xylem [CO_2_*]. Approximately 90 seconds after adding the ^13^CO_2_* solution, a sharp increase in ^13^CO_2_ efflux started and the light on the LI-6400 was turned off. Throughout the measurement period, the rate of transpiration was manipulated by switching the LI-COR desiccant between full scrub, i.e. high vapour pressure deficit (VPD) and higher rates of transpiration, and full bypass, i.e. low VPD and lower rates of transpiration. To further increase the relative humidity when the desiccant was on full bypass, a condensing tube in a water bath (VWR Scientific products, West Chester, PA, USA) was attached to the LI-COR inlet to decrease VPD to 0.5 kPa or less.

The desiccant was left on full scrub or full bypass for approximately one hour. The measurement cycle consisted of approximately 3–4 alternating high and low VPD periods. This procedure was used to gain the widest range of transpiration values possible in the dark for comparisons of transpiration and ^13^CO_2_ efflux. Data consist of four replicates for each bicarbonate concentration from four different individuals for each species.

### Anaplerotic reaction calculations

The average natural abundance of ^13^CO_2_ in the atmosphere is ~1.1% (Griffis *et al.*, 2004) and we assumed the background rate of ^13^C leaf respiration, i.e. the rate of ^13^CO_2_ respiration from an unlabeled leaf (^13^C_RL_), would be proportional to the rate of ^12^CO_2_ respiration (^12^C_RL_) according to the natural abundance for each isotopologue. Therefore, we approximated ^13^C_RL_ as 1.1% of the total respiration rate (^12^C_RL_+^13^C_RL_) and used the measured ^12^C_RL_ as the basis for calculations. This approach gave data consistent with ^12^CO_2_ and ^13^CO_2_ respiration measured from an unlabeled leaf. We calculated an expected ^13^C efflux (^13^C_cal efflux_) that would occur if all the ^13^CO_2_ added to the cut leaf exited the leaf as ^13^CO_2_. This was accomplished by multiplying the [^13^CO_2_*] in the solution fed to the leaves by the rate of transpiration and adding it to the background ^13^C_RL_. After supplying ^13^CO_2_ to the leaf, we used the ratio of observed ^13^CO_2_ efflux (^13^C_efflux_) to ^13^C_cal efflux_ to determine the fraction of supplied ^13^CO_2_ exiting the leaf and expressed it as a percentage. We estimated the rate of anaplerotic reactions by taking the difference between ^13^C_cal efflux_ and observed ^13^C_efflux_. For these calculations we assumed the transpiration rate, along with the rate of ^12^C and ^13^C effluxes were constant across one TDL/LI-COR cycle i.e. 4 or 6 minutes. We excluded the first 30 minutes after the light was turned off for these calculations to avoid potential complications from light enhanced dark respiration (LEDR) and to allow the water in the leaf to be replaced with the water supplied to the petiole.

### Stem [CO_2_^*^]

*In situ* stem CO_2_ concentration (stem [CO_2_*]) and pH were measured according to McGuire and Teskey (2002). To calibrate the CO_2_ microelectrode (GS-136CO-1 Micro Carbon dioxide electrode, Lazar Research Laboratories Inc., Los Angeles, CA, USA), four calibration tanks of 0, 0.33, 2.10, and 7.0% [CO_2_] were dissolved into Milli-Q water with a glass bubbler for at least one hour. Percent CO_2_ gas by volume in stems is the value calculated in the airspace around the xylem tissue that would exist when it is equilibrated with the total dissolved inorganic carbon in xylem sap ([CO_2_*]=[CO_2_]_aq_+ [H_2_CO_3_]+[HCO_3_^−^]+[CO_3_^2−^]). The pH of the calibration water in the glass bubblers was measured in order to calculate all forms of inorganic carbon in solution.

Insertion of the CO_2_ microelectrode was accomplished by making a hole slightly larger than the microelectrode diameter in plant stems using a drill bit. For *B. napus* a hole ~1.5 cm in diameter was drilled into the main stem with well-developed secondary tissue growth. The drill tip was inserted into the first layer of the stem, i.e. at ~0.25 cm, which was assumed to be phloem tissue, and hit a stiff inner section at ~0.50 cm, which was assumed to be the secondary xylem tissue. For *P. deltoides* the drill was placed in a woody stem that was approximately 5 cm in diameter and was drilled until it reached woody tissue at ~1 cm into the stem. For both plants, a micro pH electrode (0.1 mm immersion depth—PHR-146S micro pH electrode, Lazar Research Laboratories Inc., Los Angeles, CA, USA) was placed in the hole in the stem and the pH was measured prior to insertion of the CO_2_ microelectrode. Measurements with the CO_2_ microelectrode were recorded every minute until the mV reading was stable for 10 minutes. Following the measurement with the CO_2_ microelectrode, the pH was again measured to see if the stem sap pH had changed. Stem measurements were collected to provide data on the range of values to expect in small potted plants grown in a greenhouse. We believe these represent a minimum value for what would be found in branch tips throughout a tree canopy, if no xylem-transported CO_2_ from roots and stems arrived from outside the branch, and should be roughly similar to stem [CO_2_] in a field-grown *B. napus*. The stem [CO_2_] data were collected separately from the detached leaf measurements, using the same *P. deltoides* individuals and a separate cohort of *B. napus* plants.

### Statistical analyses

Statistical analyses were performed using R. Linear regression models were produced for rates of ^12^C_RL_ and ^13^C_efflux_ for all [^13^CO_2_*] and both species. ANOVAs were performed to detect any significant difference among [^13^CO_2_*] treatments for the rates of ^12^C_RL_ and ^13^C_efflux_ for both species. Two-way ANOVAs were used for percentage of ^13^CO_2_ exiting the leaf, estimated rate of anaplerotic reactions and ^13^C_efflux_ at a given rate of transpiration for both [^13^CO_2_*] and species. Post-hoc analysis was performed using a Tukey HSD test.

## Results

### Magnitude of xylem derived inorganic carbon efflux and retention in leaves

**Fig. 2.**
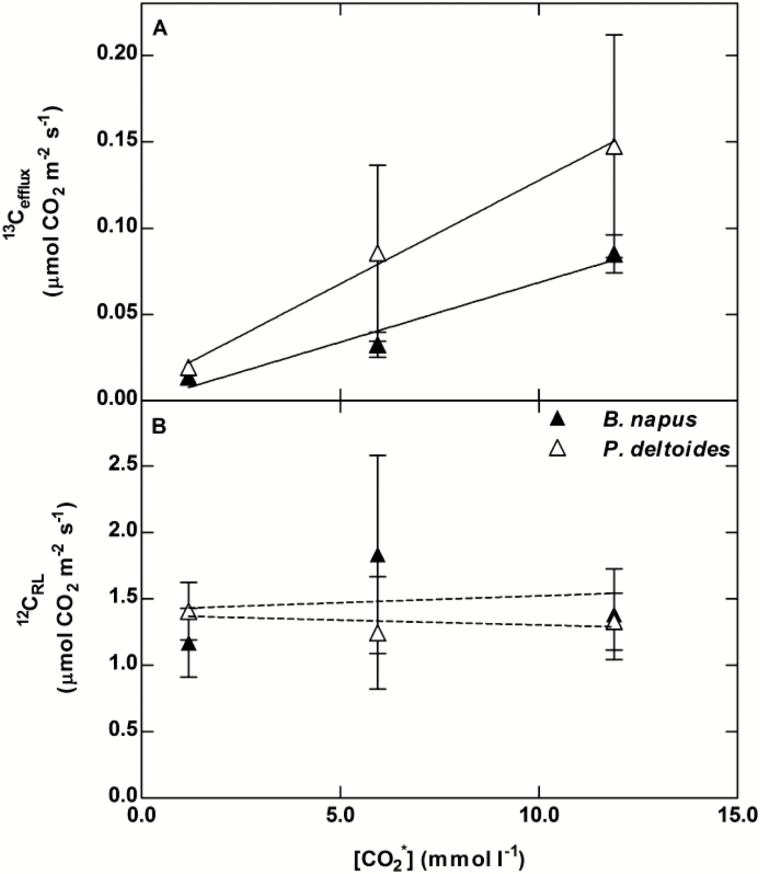
Response of gross ^13^C_efflux_ and ^12^C_RL_ in *B. napus* (closed triangle) and *P. deltoides* (open triangle) at a transpiration rate of 0.5 mmol H_2_O m^−2^ s^−1^ for each [^13^CO_2_*]. (A) Response of gross ^13^C_efflux_ to [^13^CO_2_*], R^2^ of 0.91 and 0.67 in *B. napus* and *P. deltoides*, respectively, (B) simultaneous response of ^12^C_RL_ to [^13^CO_2_*], R^2^ of <0.01 and 0.02 in *B. napus* and *P. deltoides*, respectively. Measurements were made on cut leaves of *B. napus* and *P. deltoides* placed in one of the three ^13^CO_2_* solutions. Measurements represent averages and standard deviation of four replicates at each [^13^CO_2_*].

The gross rate of ^13^C_efflux_ was dependent on the [^13^CO_2_*] supplied to the leaf when compared with a transpiration rate of 0.5 mmol H_2_O m^−2^ s^−1^ in both *B. napus* (*P*<0.001) and *P. deltoides* (*P*<0.001) (Fig. 2). Overall ^13^C_efflux_ was significantly higher in *P. deltoides* than *B. napus* (*P*=0.03) but not for pairwise comparisons at each [CO_2_*]. The gross rate of ^12^C_RL_was not significantly affected by [^13^CO_2_*] (Fig. 2B) in either *B. napus* (*P*=0.80) or *P. deltoides* (*P*=0.72).

**Fig. 3.**
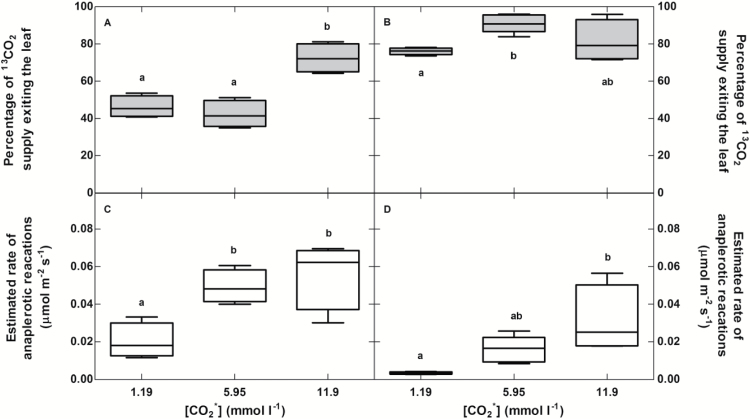
The percentage of gross ^13^CO_2_* exiting the leaf in the transpiration stream in (A) *B. napus* and (B) *P. deltoides* across three different [^13^CO_2_*]. Estimated rate of anaplerotic reactions, i.e. retention of ^13^C in the dark, in (C) *B. napus* and (D) *P. deltoides* across three different [^13^CO_2_*]. Measurements represent averages and standard deviation of four replicates.

The ^13^CO_2_* provided to the petioles entered the darkened leaves through the xylem via the transpiration stream and exited the leaves as ^13^CO_2_ (Fig. 3A, B). Approximately 50% of ^13^CO_2_* at 1.19 mmol l^−1^ and 5.95 mmol l^−1^ and ~70% at 11.9 mmol l^−1^ exited the leaf as ^13^CO_2_ in *B. napus*. In *B. napus* the percent of ^13^CO_2_* exiting the leaf at 11.9 mmol l^−1^ was significantly higher than at both 1.19 mmol l^−1^ (*P*<0.001) and 5.95 mmol l^−1^ (*P*<0.01) (Fig. 3A). The percentage of ^13^CO_2_* exiting the leaf as ^13^CO_2_ for *P. deltoides* was ~80% across all bicarbonate concentrations (Fig. 3B). It was significantly different between 1.19 mmol l^−1^ and 5.95 mmol l^−1^ (*P*<0.05) but not between other concentrations (Fig. 3B). The percent efflux was higher overall in *P. deltoides* than *B. napus* (*P*=0.001), though not for the pairwise comparison at a high [CO_2_*] (*P*=0.43) (Fig. 3A, B).

The estimated retention rate of ^13^CO_2_* by an individual leaf was similar across the higher [^13^CO_2_*] in *B. napus* (~0.05 μmol CO_2_ m^−2^ s^−1^) and was slightly lower in the lower [^13^CO_2_*] (~0.02 μmol CO_2_ m^−2^ s^−1^) (Fig. 3C). The estimated retention rate was significantly different between 1.19 mmol l^−1^ and 5.95 mmol l^−1^ (*P*<0.05) and 11.9 mmol l^−1^ (*P*<0.01) in *B. napus*. The retention rate increased between the lowest and highest [^13^CO_2_*] for *P. deltoides* (~0.01 μmol CO_2_ m^−2^ s^−1^ to ~0.03 μmol CO_2_ m^−2^ s^−1^) (*P*<0.01) (Fig. 3D); however, differences between the other concentrations were not significant. The retention rate differed overall between species (*P*<0.001) but did not show any consistent differences with respect to [CO_2_*]. They were only significantly different at 5.95 mmol l^−1^ (*P*<0.005, using pairwise comparisons).

**Fig. 4.**
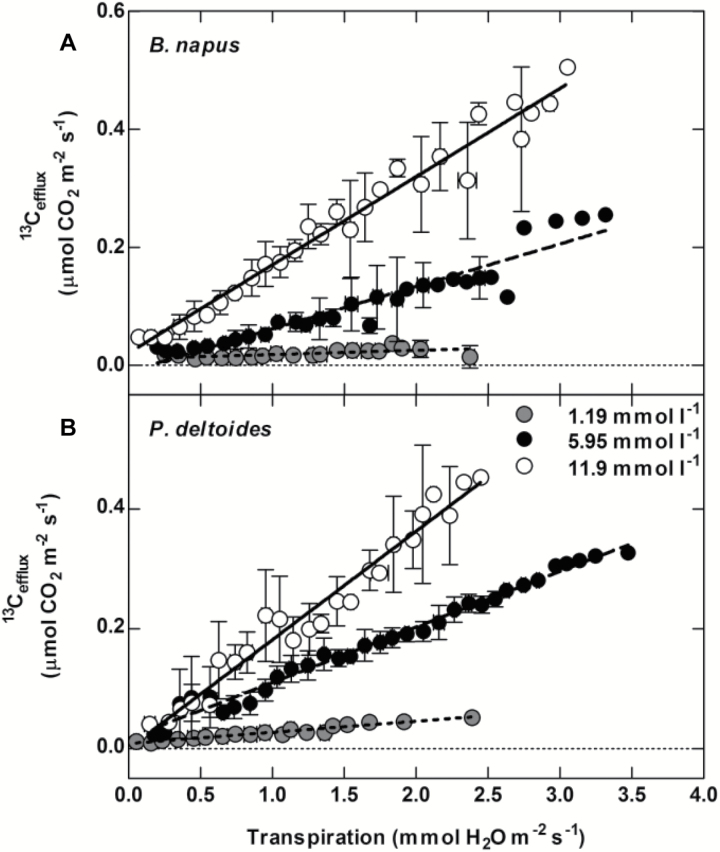
Transpiration dependence of gross ^13^C_efflux_ for three [^13^CO_2_*] measured with the TDL. (A) *B. napus* and (B) *P. deltoides*. In both panels grey circles represent 1.19 mmol l^−1^ [^13^CO_2_*] (*B. napus* y=0.008x+0.001, R^2^=0.68; *P. deltoides* y=0.02x +0.01, R^2^=0.98), closed circles are 5.95 mmol l^−1^ [^13^CO_2_*] (*B. napus* y=0.07x−0.01, R^2^=0.82; *P. deltoides* y=0.09x+0.02, R^2^=0.98), and open circles are 11.9 mmol l^−1^ [^13^CO_2_*] (*B. napus* y=0.15x+0.02, R^2^=0.97; *P. deltoides* y=0.18x +0.00, R^2^=0.93). Measurements were made on excised leaves of *B. napus* and *P. deltoides* placed in one of the three [^13^CO_2_*] solutions. Measurements represent averages and standard deviation of four replicates. Transpiration was averaged over 0.1 mmol H_2_O m^−2^ s^−1^ increments.

**Fig. 5.**
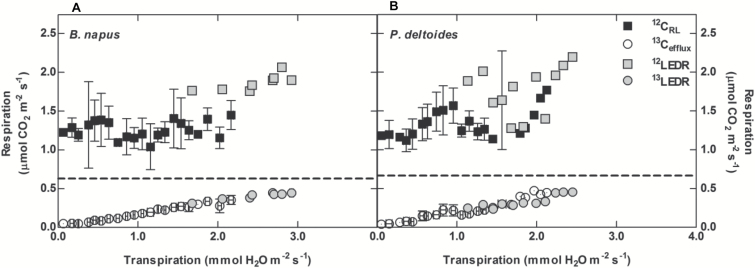
Comparison of leaf respiration (^12^C_RL_, closed squares) and gross ^13^C_efflux_ (open circles) (A) *B. napus* and (B) *P. deltoides* leaves fed with 11.9 mmol l^−1 13^CO_2_*. ^12^LEDR (grey squares) and ^13^LEDR (grey circles) represent the light enhanced dark respiration rate i.e. during the first 30 minutes after the light was turned off). The dotted line represents half the rate of leaf respiration, observed in (Fig. 2) of 0.63 μmol±0.12 CO_2_ m^−2^ s^−1^ and 0.67 ± 0.11 μmol CO_2_ m^−2^ s^−1^ in *B. napus* and *P. deltoides*, respectively. Measurements represent averages and standard deviation of four replicates. Transpiration was averaged over 0.1 mmol H_2_O m^−2^ s^−1^ increments.

Transpiration decreased rapidly after turning off the light. This generated a range of transpiration rates where we could examine the relative magnitudes of ^12^C_RL_ and ^13^C_efflux_ (Fig. 4, 5). The gross rate of ^13^C_efflux_ was dependent on both the [^13^CO_2_*] supplied to the xylem (*P*<0.005) (Fig. 4) and transpiration rate (*P*<0.005) (Fig. 4, 5). In contrast, gross ^12^C_RL_ did not significantly correlate with the transpiration rate in *B. napus* (*P*=0.54) or *P. deltoides* (*P*=0.74) (Fig. 5). Gross ^12^C_RL_ was significantly higher during the first 30 minutes of the dark period where LEDR occurs in both *B. napus* and *P. deltoides* (Fig. 5) (*P*<0.005). As expected, the LEDR response did not affect the gross ^13^C_efflux_ in either *B. napus* or *P. deltoides* (*P*<0.0001) (Fig. 5). We also found that ^13^C_efflux_ approached half that of ^12^C_RL_ in both species during our experiments. The rate of dark respiration when excluding LEDR was 1.26 ± 0.23 μmol CO_2_ m^−2^ s^−1^ and 1.33 ± 0.21 μmol CO_2_ m^−2^ s^−1^ in *B. napus* and *P. deltoides*, respectively. Linear regressions, R^2^=0.97 for *B. napus* and R^2^=0.93 for *P. deltoides*, show that for leaves supplied with 11.9 mmol l^−1^ [^13^CO_2_*], the gross efflux of xylem-transported CO_2_ will equal ½R_d_ at a transpiration rate of 4 mmol H_2_O m^−2^ s^−1^ and 3.69 mmol H_2_O m^−2^ s^−1^ for *B. napus* and *P. deltoides*, respectively (Fig. 5). At all [^13^CO_2_*] for both species, gross ^13^C_efflux_ was undetectable when transpiration was <0.1 mmol H_2_O m^−2^ s^−1^.

### Stem [CO_2_^*^]

In greenhouse grown plants, the average [^13^CO_2_*] in the stem was 0.7 ± 0.2 mmol l^−1^ and 2.8 ± 3.5 mmol l^−1^ for *B. napus* and *P. deltoides*, respectively. Stem pH in *P. deltoides* ranged between 5.1 and 6.4, with an average of 5.9 ± 0.3. Stem pH in *B. napus* ranged between 5.3 and 6.1, with an average of 5.7 ± 0.3.

## Discussion

### Magnitude of xylem derived inorganic carbon efflux

Our experimental approach allows simultaneous measurements of the gross efflux of xylem-transported ^13^CO_2_* exiting leaves as ^13^CO_2_ with transpiration, as well as the efflux of ^12^CO_2_ from leaf respiration in real time. We applied this approach to excised leaves in order to eliminate complications from radial diffusion through stems and branches. We also focused our data collection on the dark conditions immediately after the light was turned off, in order to compare CO_2_ efflux with a wide range of transpiration rates, from typical night-time rates through to the low end of typical daytime rates. We demonstrated that transport of labeled ^13^CO_2_* in xylem was highest when both [^13^CO_2_*] (Fig. 2) and transpiration (Fig. 4) were high. The broad range of transpiration values also allowed us to generate a robust relationship between gross efflux of xylem-transported ^13^CO_2_* (^13^C_efflux_) and transpiration that can be used to predict ^13^C_efflux_ in conditions where transpiration rate and xylem [CO_2_*] are known. Considering the very different petiole and leaf anatomies of *P. deltoides* and *B. napus*, we were surprised at how similar this relationship was between species (Fig. 4).

When [CO_2_*] in stems is high, i.e. 11.9 mmol l^−1^, and when transpiration rates are high, i.e. >3 mmol H_2_O m^−2^ s^−1^, our estimates indicate the gross efflux of xylem-transported CO_2_* approaches half the rate of leaf respiration (Fig. 5). This efflux could therefore account for some of the variation observed in measurements of leaf respiration in the light. Measurements of stem [CO_2_*] in field grown *P. deltoides* have been reported to range from the low end, between 2.8–9.5 mmol l^−1^ (Saveyn *et al.*, 2008), to the high end, between 20 and 35 mmol l^−1^ (Aubrey and Teskey, 2009), while reported transpiration rates in *P. deltoides* ranged from 3.4 mmol H_2_O m^−2^ s^−1^ (Bassman and Zwier, 1991) to 4.5 mmol H_2_O m^−2^ s^−1^(Barron-Gafford *et al.*, 2007). This indicates our selection of [^13^CO_2_*] were within the range of available data for *P. deltoides*. However, it should be noted that measurements of [^13^CO_2_*] in *P. deltoides* are on the high end of reported tree values (Teskey *et al.*, 2008).

Most leaves are attached to small branches on trees or to the relatively small stems and branches of herbaceous plants, where [^13^CO_2_*] has not been well characterized. Models of [CO_2_*] distribution throughout trees predict it increases with distance from the ground since CO_2_ is generated at higher rates than it diffuses out of a tree or shrub (Hölttä and Kolari, 2009). These predictions were supported by measurements in *Quercus pyrenaica* (Salomon *et al.*, 2016*a*). Hölttä and Kolari (2009) also modeled that [CO_2_*] declines as the stem tapers towards the top of the tree and may only end up at around 5% of the basal [CO_2_*] at the leaf level. Surprisingly, no study has measured [CO_2_*] in the main stem near tree bases simultaneously with terminal branch data in order to validate these model predictions. Furthermore, there is almost no information on xylem [CO_2_*] in small tree branches or herbaceous plants. Stringer and Kimmerer (1993) found that terminal branch measurements of xylem [CO_2_*] in large *P. deltoides* trees were between 0.5 mmol l^−1^ and 0.9 mmol l^−1^ when growing in well drained soils and up to 9.4 mmol l^−1^ in water logged soils. Our small potted plants grown in a greenhouse contained 0.7 ± 0.2 mmol l^−1^ and 2.8 ± 3.5 mmol l^−1^ [CO_2_*] for *B. napus* and *P. deltoides*, respectively. This is on the low end of our experimental design and may be a reasonable value for similarly sized plants in the field. However, terminal branches of mature trees would also contain high [CO_2_*] transported from the respiration of larger branches, stem, and even roots. Modeling the effect of the rest of the tree is not currently possible because there is very little data on long-term, namely diurnal and seasonal, variation in stem [CO_2_*] in mature trees. What data do exist show high variability (Etzold *et al.*, 2013; Salomón *et al.*, 2016*b*). Using our approach to calculate gross efflux rates of xylem-transported [CO_2_*] arriving at a leaf would therefore also require measurement of the [CO_2_*] in the branches near the leaf, as well as the rate of transpiration.

### Retention of xylem-transported CO_2_ in darkened leaves

At high [^13^CO_2_*], the percentage of xylem-transported ^13^CO_2_* exiting the leaf was fairly high for both species (Fig. 3) and near the 83% Stringer and Kimmerer (1993) observed when they supplied 1 mmol l^−1 14^CO_2_* to *P. deltoides* leaves. However, at lower [^13^CO_2_*] the percentage ^13^CO_2_* exiting the leaf was reduced in *B. napus* (*P*=0.001). As these results show, all ^13^CO_2_* entering the leaf did not exit as ^13^CO_2_ during our measurements, even though leaves were darkened during labeling. We therefore used a mass balance approach to determine the rate of retention of ^13^CO_2_* supplied to the leaf to facilitate comparison with other fluxes. Interestingly, the retention rate only gradually increases with higher [^13^CO_2_*] supplied (Fig. 3C, D). We hypothesize that this retention represents the rate that anaplerotic reactions consumed the supplied ^13^CO_2_*. Our estimated rate of retention by anaplerotic reactions for *P. deltoides* at 1.19 mmol l^−1^ [^13^CO_2_*], 0.003 μmol m^−2^ s^−1^, is similar to the rate (0.002 μmol m^−2^ s^−1^) calculated from data by Stringer and Kimmerer (1993) for leaves supplied with 1 mmol l^−1^ [^14^CO_2_*]. However, our estimated rate of anaplerotic reactions is only about 1% of the rate of phosphoenolpyruvate carboxylase (PEPC) activity in previously published studies with *P. deltoides* plants exposed to ozone or varying light treatments (Loreto *et al.*, 2007).

We also calculated a significantly higher overall apparent rate of retention in *B. napus* than in *P. deltoides* (*P*<0.001), though not for pairwise comparisons (Fig. 3). It is possible that the thick, green petioles and the blade of the *B. napus* leaf have slightly higher rates of anaplerotic respiration and that *B. napus* petioles have little higher radial efflux of CO_2_ than the thin, waxy petioles of *P. deltoides*. Measuring how much of the retained ^13^C is acid stable, which parts of the leaf have label and what compounds are labeled will be necessary to fully determine the fate of xylem-transported CO_2_*. If petiole efflux of ^13^CO_2_ is large, we would expect that the percentage of ^13^CO_2_ exiting the leaf would decrease with increasing [^13^CO_2_*] due to a higher concentration gradient between the petiole and the atmosphere. However, the percentage of ^13^CO_2_ exiting the leaf does not show consistent patterns with the [^13^CO_2_*] supplied to the leaf.

### Implications for understanding leaf and stem carbon fluxes

Leaf efflux of xylem-transported CO_2_* is controlled by the rate of transpiration and the xylem [CO_2_*] (Fig. 4) but this is not accounted for when characterizing leaf respiration. We believe a significant amount of the uncertainty in plant respiration could be due to limited efforts to quantify the effects of xylem-transported CO_2_*. There is potential for further characterization to improve our understanding of a wide range of leaf-level processes that have been hard to quantify such as: gross photosynthesis, the extent to which day respiration is down regulated in the light (Atkin *et al.*, 2000; Hurry *et al.*, 2005; Ayub *et al.*, 2011), the response of respiration to stressors such as drought (Ayub *et al.*, 2011; Rowland *et al.*, 2015), the acclimation to environmental conditions such as temperature (Vanderwel *et al.*, 2015), night respiration for species whose night-time transpiration is high and/or variable (Snyder *et al.*, 2003; Resco de Dios *et al.*, 2015), variation in the acclimation rates of respiration and photosynthesis to temperature between plant functional types (Campbell *et al.*, 2007), the amount of CO_2_ recycling in leaves including the potential recycling of xylem-transported CO_2_* (Busch *et al.*, 2013), the diffusion of CO_2_ in leaves (Evans and von Caemmerer, 2013), and the activity of alternative decarboxylations associated with photorespiration (Cousins *et al.*, 2008; Cousins *et al.*, 2011).

Essentially, any measurement of leaf respiration that induces or includes changes in transpiration is susceptible to this error for species where xylem [CO_2_*] is high (Fig. 1). For example, the efflux of xylem-transported CO_2_* may interfere with methods for measuring day respiration in leaves. The two most common methods require varying photosynthesis at low light or low [CO_2_] (Kok, 1949; Laisk, 1977). These measurements are time consuming and require highly accurate measurements of small fluxes that would be compromised if transpiration varies during the measurement, for example due to light or CO_2_ affecting stomatal aperture. Measurements of leaf photosynthesis are also directly affected since some of the xylem-transported CO_2_* is recycled by leaves (Stringer and Kimmerer, 1993; McGuire and Teskey, 2002; Bloemen *et al.*, 2013*a*,b; Bloemen *et al.*, 2015*b*) and model assumptions would be violated if some escaped the leaf without passing through chloroplasts (von Caemmerer, 2013).

In addition, any changes in root, stem, or branch respiration occurring during the measurement of small leaf fluxes would cause errors in leaf respiration when transpiration is sufficient. There are significant uncertainties surrounding the efflux of CO_2_ from stems, as well as the rate of anaplerotic reactions, and the effects of both on the measurement of stem respiration (Teskey *et al.*, 2008; Bloemen *et al.*, 2015*a*). These uncertainties include diurnal variation in stem and root respiration, which is well documented but poorly understood, showing some linkage to diurnal patterns of carbon supply from shoots as well as other drivers (Bloemen *et al.*, 2015*b*; Snell *et al.*, 2015; Steppe *et al.*, 2015). Our method of adding labeled bicarbonate to leaves could be applied to stems to improve estimates of the gross efflux of CO_2_ and the rates of gross stem photosynthesis in real time, by helping to partition the rates of radial efflux through the stem relative to the vertical transport of CO_2_* away from the site of respiration.

### What would it take for this flux to be significant globally?

Although we have point data on stem [CO_2_*] for over two dozen tree species (Teskey *et al.*, 2008), we still need more data on the relationship between stem and branch tip [CO_2_*] as well as their diurnal and seasonal patterns before we can make a solid prediction of what the contribution is from the ~3 trillion trees on Earth (Crowther *et al.*, 2015). However, if [CO_2_*] averages near 1 mmol l^−1^ at the point where petioles attach to the stem or branch, which is a common value on the low end reported for tree stems (Teskey *et al.*, 2008), and if average transpiration rates are around 0.5 mmol H_2_O m^−2^ s^−1^ i.e. much closer to night-time than daytime rates (see Rescos de Dios *et al*. 2015 for regulation of night-time transpiration), then our data (Fig. 2) and those from Stringer and Kimmerer (1993) predict the efflux to be just over 1.5% of respiration. At the global scale, an error in respiration this size would account for a large portion of the error in global carbon cycle models (Hanson *et al.*, 2016).

## Conclusions

Using our novel online TDL gas exchange method paired with ^13^CO_2_* labeling, we found that when xylem [CO_2_*] and transpiration are high, the efflux of xylem-transported CO_2_* could cause large errors in observed leaf respiration. This would be most likely in the daytime when stomata are open, complicating the measurement of day respiration. We observed a fairly similar efflux of xylem-transported CO_2_* from *P. deltoides* and *B. napus* leaves, which suggests the effect of leaf and petiole morphology on the efflux is modest. However, data from many more species that include the diurnal and seasonal patterns of xylem [CO_2_*] in branch tips, where leaves attach, are needed to ascertain how often xylem-transported CO_2_* interferes with measurements of leaf respiration or estimations of regional or global carbon fluxes. Future work with this approach will allow examination of the dynamics of the rate of xylem [CO_2_*] transport, radial efflux and internal re-fixation by stem, branches and leaves, relative to rates of respiration and photosynthesis occurring in each organ.

## Abbreviations:

[CO_2_*]: total inorganic carbon concentration
LEDR: light enhanced dark respiration
stem [CO_2_*]: *In situ* stem CO_2_ concentration
TDL: tunable diode laser
VPD: vapour pressure deficit.
